# Comparison of the Diarrhœic Stools and Accompaniments, of Bryonia, Natrum Sulphuricum, and Oxalic Acid

**Published:** 1885-09

**Authors:** George H. Clark

**Affiliations:** Germantown


### COMPARISON OF THE DIARRHŒIC STOOLS AND ACCOMPANIMENTS, OF BRYONIA, NATRUM SULPHURICUM, AND OXALIC ACID.

George H. Clark, M. D., Germantown.

Bryonia stools are brown, thin, fecal, while Nat. sul. has *thin, yellow, fluid,* and *yellowish-green ;* Ox. ac. stools are brown, fecal, and muddy; also constant involuntary stools; while Bry. has frequent stools and involuntary, but the involuntary are during sleep; Nat. sul. stools are only occasionally involuntary while passing flatus or urine or during sleep; the stools of the latter are not frequent, those of Bry. are. Bry. stools are acrid, causing soreness of the anus; are thin, bloody; *copious, pasty,* and *dark green;* like dirty water, with a whitish, granulated sediment of undigested food. They are putrid, smelling like rotten cheese, and alternate with constipation. Nat. sul. stools are painless, watery, with much flatus. Under Ox. ac. there is a continuous discharge of white mucus.

Bry. diarrhœa is aggravated *in the morning,* about 2 or 3 A.M.; *on first rising and moving about; from moving even hand or foot.* Nat. sul. agg. *in morning, after rising and moving about.* Ox. ac. agg. in morning, after breakfast, and from motion (pains). Bry. is also at night; worse in hot weather; *whenever the weather gets warmer.* Nat. sul. is worse in cold evening air; after a spell of damp weather; and from living in damp houses; also after farinaceous food. Bry. is worse after taking cold; after cold drinks; from milk; from eating stewed fruit or vegetables; after eating sauerkraut; from anger and chagrin; and from sitting up. Under Ox. ac. the agg. is as soon as one drinks coffee; as soon as he lies down the diarrhœa returns.

With Bry. the amel. is *by keeping still, by doubling up, or by lying on the abdomen.* Nat. sul. symptoms are generally ameliorated after breakfast and in the open air; while Ox. ac. is better from rest (pains).

*Before the stool:* Bry. has colic; cutting pains; nausea. Nat. sul. has contractive pain in abdomen, extending into the chest; pains in the groins and hypogastrium. Violent colic and rumbling. Ox. ac. has headache. Twisting colic around umbilicus.

*During stool:* Bry. has burning at anus; prolapsus ani; vomiting; thirst; drowsiness; chilliness; offensive flatus. Nat.

sul. has burning at anus and slight tenesmus; *profuse emission of flatus;* voluptuous feeling. Ox. ac. has colic about umbilicus; colicky pains seem to radiate from a small spot. Violent urging; griping pains in anus, so severe as to cause headache and heat in head.

*After stool:* Bry., heat and drowsiness; Nat. sul., burning at anus; relief of colic; cheerfulness. Ox. ac., nausea; tension in the calves; dryness in throat; relief of pain in small of back.

Then of much importance are the accompanying general symptoms. Without these one cannot be sure of the proper remedy, and no picture of the case is complete without them. Bry. has a desire for things which do not exist, or which are refused when offered. Peevishness; ill-humor; delirium. *Desire to get out of bed and go home. Talking of the business of the day. Head hot, with frequent raising of hands to the head.* Boring of head back into pillow, or rolling from side to side. Eyes glassy and staring; sleeps with eyes half-open. Sensitiveness to noise and light. Dry, swollen, and cracked lips. *Mouth so dry that child will not nurse until it is moistened.* Tongue dry and red or brown, or white, or yellow. THIRST FOR LARGE QUANTITIES AT LONG INTERVALS. *Bitter taste in mouth and of food. Nausea and fainting on sitting up.* Desire for cold drinks (yet cold drinks agg.), wine, and coffee, and sour drinks. Vomiting of bitter substances, of yellow, green mucus. Pain in bowels after eating or drinking. Urine dark-red and clear. Desire to lie down and remain quiet.

Nat. sul.: *Thirst in the evening;* sour risings with heartburn. Bitter taste. Copious formation of gas, causing distention of abdomen and flatulent colic. *Incarceration of flatulence,* especially in ascending colon and signoid flexure. Colic is particularly worse before breakfast, when the stomach is empty; relieved by kneading the abdomen and by borborygmus. Bruised pain in the intestines, *stitches in region of liver, and sensitiveness when walking in open air.* Liver is *swollen and sore to touch or to any jar of body.* Constant uneasiness in bowels and urging to stool. *Passing of large quantities of flatus, mostly fetid.* Constant desire to take deep, long breath. This remedy is frequently indicated in chronic diarrhœa, where the loose morning stool is the leading symptom. It is particularly indicated where there is inflammation and suppuration around the roots of the nails (panaritium) or a tendency thereto. Ox. ac.: *Thinking of the symptoms* aggravates them. Exhilaration. Stomach very sensitive to pressure. Frequent pains and soreness about the umbilicus. Copious urine.

